# Sleep-inducing algorithms: can artificial intelligence help shiftworkers and those working nonstandard hours sleep better?

**DOI:** 10.1093/sleepadvances/zpag015

**Published:** 2026-01-28

**Authors:** Ruby G Smith, Grace E Vincent, Madeline Sprajcer, Sally A Ferguson, Dean J Miller, Corneel Vandelanotte

**Affiliations:** Appleton Institute, School of Health, Medical, and Applied Sciences, Central Queensland University, Adelaide, South Australia, Australia; Appleton Institute, School of Health, Medical, and Applied Sciences, Central Queensland University, Adelaide, South Australia, Australia; Appleton Institute, School of Health, Medical, and Applied Sciences, Central Queensland University, Adelaide, South Australia, Australia; Appleton Institute, School of Health, Medical, and Applied Sciences, Central Queensland University, Adelaide, South Australia, Australia; Appleton Institute, School of Health, Medical, and Applied Sciences, Central Queensland University, Adelaide, South Australia, Australia; Appleton Institute, School of Health, Medical, and Applied Sciences, Central Queensland University, Adelaide, South Australia, Australia

**Keywords:** behavioral sleep medicine, circadian rhythms, consumer sleep technology, machine learning, shift work, sleep hygiene

## Abstract

Individuals working nonstandard hours face a range of negative health, safety, and productivity outcomes, largely driven by sleep disruptions associated with their schedules. Existing interventions to improve sleep often adopt a one-size-fits-all approach, overlooking the diversity of individual needs, preferences, and work contexts. These factors are critical considerations for any intervention aiming to improve the sleep of individuals working nonstandard hours as schedules can differ dramatically, both between individuals and within an individual’s schedule. Advances in wearable consumer sleep technology and artificial intelligence, like the use of reinforcement learning and large language models, now offer the opportunity for highly tailored just-in-time-adaptive-interventions (JITAIs), or digital interventions that adapt to individuals’ unique contexts to provide personalized, timely behavioral support. This paper proposes that integrating artificial intelligence and wearable consumer sleep technology with JITAIs has the potential to deliver the right support, at the right time, and in the right context for each individual nonstandard-hour worker. By directly responding to the unpredictable and variable hours these workers face, such technologies could set a new standard for personalized health, safety, and productivity interventions. Challenges associated with incorporating artificial intelligence and wearable consumer sleep tracking devices into JITAIs, such as trust, technological and algorithmic inaccuracies, user engagement, and cost, are also discussed as key considerations for successful implementation.

*This paper is part of the Consumer Sleep Technology Collection*.

## Working Nonstandard Hours and the Associated Health, Safety, and Productivity Impacts

Individuals working nonstandard hours, or those who work outside standard 0900–1800 daytime working hours [1], make up a significant proportion of the global workforce. In Australia, approximately 14.5% of the workforce are shiftworkers, 19% have on-call work arrangements, and 32% usually work overtime hours [2]. These numbers align closely with those of other nations globally. In the United States (US), shiftworkers make up approximately 16.4% of the workforce [3] and in the United Kingdom (UK) 27% of the workforce regularly work night shifts [4]. Society relies on nonstandard-hour workers for the delivery of a wide range of essential services across industries required to operate around the clock [2]. Additionally, increasing market globalization has led to reliance on nonstandard-hour workers to maintain the production and delivery of goods and services across all hours of the day [5]. These nonstandard-hour workers include shift-, on-call-, and gig-based workers [6, 7].

Individuals working nonstandard hours face a range of negative health and safety outcomes [1, 8, 9], driven by physiological disruptions [10, 11], with subsequent costs for individuals and society [12]. High variability in shift schedules, i.e., a combination of morning, night, and/or afternoon shifts, often leads to circadian rhythms becoming misaligned, causing a reduction in sleep duration and quality [8, 10, 13, 14]. Studies have shown strong evidence supporting associations between shiftwork and increased risk of cardiovascular disease [15], immune dysfunction [16], hypertension [17], diabetes mellitus [18], some types of cancer [19], and a range of negative mental health outcomes, particularly depression [20]. The sleep impacts of working nonstandard hours lead to a reduction in alertness and productivity, which can increase lapses in judgment [5]. As a result, nonstandard-hour workers are nearly twice as likely to make an error, sustain injuries at work, or have an incident at work as those working standard hours [21–23]. Furthermore, absenteeism and staff attrition rates are more than double in shiftwork settings [24, 25]. Improving these workers’ sleep is critical to reducing health and safety risks and maintaining efficient operations. However, the high variability in their schedules makes it challenging to develop tailored behavior change interventions.

### How can we use behavioral interventions to improve sleep in individuals working nonstandard hours?

Behavioral sleep interventions such as individual coaching and sleep hygiene interventions are effective for standard-hour workers [26, 27] but largely ineffective for shift, on-call, and gig workers [28–30]. This limited efficacy extends across digital and face-to-face delivery methods, and likely reflects the difficulty of generalized sleep interventions in accommodating the complex and highly variable sleep–wake schedules of these workers [30]. Further, generalized sleep interventions underpinned by standard sleep hygiene guidelines may conflict with fatigue management advice, such as utilizing daytime napping and consumption of caffeine [31]. These strategies can be highly beneficial for workers on nonstandard hours, and discouraging their use may partly explain why generalized sleep interventions often fall short for this group [28, 32]. Some interventions designed specifically for individuals working nonstandard hours have improved sleep duration and quality [29]. However, tailoring behavioral support to each individual worker requires interventions such as individual coaching, which are difficult to deploy at scale.

Just-in-time adaptive interventions (JITAIs) are technology-supported interventions that deliver personalized support at the most ideal time, and have the potential to account for variable schedules [33–35]. JITAIs have already been used successfully to support health behavior change [35], including sleep. For example, a pilot study of an evidence-based mobile health JITAI was conducted, which aimed to improve sleep in military service members [36]. Results showed that 70% of participants met treatment response criteria (significantly improved Insomnia Severity Index scores) at 6 weeks post-treatment [36]. However, the intervention relied on participants accessing a “clinician portal,” where trained clinicians offered individualized advice [36]. The results suggest that a mobile JITAI to support improved sleep of workers with variable hours has promise. However, the feasibility of the widespread roll-out of such an intervention, where each worker has access to a trained clinician offering real-time sleep advice, may be limited. Future interventions that integrate wearable sleep technology and artificial intelligence (AI) or machine learning in sleep interventions for nonstandard-hour workers are an alternative potential solution.

### Wearable consumer sleep technology

Consumer sleep technologies are devices and apps that aim to measure sleep and are available to the public for purchase without a prescription [37]. Wearable consumer sleep tracking devices are a kind of consumer sleep technology worn on the body, like smartwatches, wristbands, or rings [38]. These devices are becoming increasingly advanced in their ability to measure sleep, with technologies such as accelerometry, electroencephalography (EEG) sensors measuring brain activity, and pulse oximeters incorporated into consumer-grade wearables. As a result, wearable consumer sleep tracking devices are approaching levels of accuracy in measuring sleep previously achievable only via clinical-grade sleep technologies [38]. A recent investigation compared the accuracies of five widely used consumer sleep tracking devices with research-grade actigraphy [39]. While devices did not measure sleep stages with comparable accuracy, all devices except one measured total sleep duration at a comparable level of accuracy to research-grade actigraphy [39]. Furthermore, the WHOOP wearable has been compared to polysomnography, the gold-standard measure of sleep, with results showing 86–90% agreement on categorization of sleep/wake cycles [40]. Additionally, the latest validation study of the Oura ring revealed 91.7–91.8% accuracy in categorization of sleep/wake cycles when compared with polysomnography [41]. These results show promise regarding the ability of wearable consumer sleep tracking devices to accurately measure sleep-wake cycles and the potential to soon measure sleep staging. These levels of accuracy would provide valuable insights to inform behavioral sleep interventions for individuals working nonstandard hours.

Wearable consumer sleep tracking devices are currently being used in some behavioral sleep interventions. A systematic review found that wearable consumer sleep tracking devices are primarily used to deliver insomnia treatments and to improve general sleep health as part of wellness programs [42]. Interventions using wearable consumer sleep tracking devices as part of interventions to improve sleep duration have shown some promising preliminary results [38, 43, 44]. Recent advancements now allow for increasingly accurate real-time sleep health insights [45], which may be precise enough to inform JITAIs designed to improve sleep in individuals working nonstandard hours. Accuracy of wearable sleep trackers is important for the design of a JITAI for nonstandard-hour workers because their sleep-wake schedules are subject to last-minute changes and high variability (both for individuals and between individuals). If wearable sleep trackers are unable to detect things such as a worker being “called in” while winding down for sleep, a JITAI may provide unhelpful advice. Accurate, real-time data received from wearable sleep trackers could mitigate the challenge of high variability and unpredictability in sleep-wake schedules when it comes to designing sleep interventions for the various types of nonstandard-hour work. Mitigating the challenge of high variability and unpredictability in shift schedules would allow practitioners or software developers to provide highly tailored sleep advice in real time for each worker.

Real-time data from consumer sleep tracking devices is already being combined with preprogrammed algorithms to deliver behavior change support, but these preprogrammed algorithms are restricted. For example, a micro-randomized trial of a JITAI that delivered personalized feedback based on the previous night’s wearable sleep data was conducted [46]. Results showed that the intervention increased participants’ sleep duration by an average of 40 minutes per night [46]. While this methodology may be effective for standard-hour workers, pre-programmed rule-based (If–Then) decision-making restricts these interventions to the specific “contexts” in which they were originally trained [33]. Thus, developers must train a JITAI to “decide” what type of support to offer each individual, depending on the situation, by programming rules for every possible scenario [33]. For example, in a JITAI to improve sleep, a rule might be: *If* the previous night’s sleep was <6 hours, *Then* provide a cue to improve tonight’s sleep duration. Developers can also tailor interventions based on contextual factors such as location, weather, and step count data [33]. For instance, a JITAI for sleep may cue a user to begin winding down when their location changes from “work” to “home.”

Individuals working nonstandard hours require further personalization than preprogrammed algorithms can provide. While a cue to wind down upon returning home may sometimes be useful, it does not account for critical variables such as the timing of the next shift or a user’s stress and energy levels post-shift. These insights are crucial to inform a JITAI for individuals working nonstandard hours because they often change and preprogramming to account for them is difficult. Because it is not feasible to preprogram algorithms for the constantly changing sleep and work arrangements of individuals working nonstandard hours, more advanced approaches such as AI are needed to deliver appropriately tailored sleep advice. This paper argues that the integration of AI and wearable consumer sleep tracking devices enables the delivery of JITAIs capable of providing highly personalized, context-dependent sleep advice at scale to individuals working nonstandard hours.

### Wearable consumer sleep technology paired with artificial intelligence and machine learning

AI is increasingly used in sleep medicine and digital health to enhance clinical practice, support research, and deliver tailored, on-demand behavior change interventions [47–49]. Machine learning can be a type of AI and is particularly promising [49] due to its ability to analyse and learn from large datasets before making predictions regarding future behavior [50]. These predictions can then inform personalized and contextually relevant behavioral support. For example, machine learning algorithms trained with accelerometer data from 80 811 UK adults accurately predict sleep efficiency and identify the best pre-bed opportunities to provide sleep advice [51]. This represents a significant leap forward from current interventions underpinned by static rules-based (If–Then) decision making. However, more advanced subtypes of machine learning and AI, like reinforcement learning, k-means clustering, random forests and deep learning, natural language processing, and large language models (LLMs) allow for increased personalization [52] and more engaging delivery methods [53].

Reinforcement learning, a subtype of machine learning [54], “learns” the most effective action in a given context through trial and error [55, 56]. In the context of a sleep intervention, improved sleep acts as a reward for the algorithm and results in increased use of the action(s) that prompted it. Conversely, if an action results in poorer sleep, this acts as a penalty to the algorithm, and the ineffective prompts would be phased out. Reinforcement learning allows for digital behavior change interventions to improve in efficacy for each individual over time, and helps to mitigate the issue of heterogeneity between individuals’ differing responses to different actions [55]. In addition to improving over time for each individual, reinforcement learning is able to improve at the group level over time by learning what works well and what does not in certain cohorts [56]. For example, a reinforcement learning algorithm can learn what works best for different groups, such as younger versus older adults, and tailor its approach accordingly. In one study, participants who received messages that became increasingly personalized over time through reinforcement learning increased their daily step counts by 19%, compared with only 1.6% in participants who received randomized motivational messages [57]. Recent advancements in machine learning technologies have further enhanced the ability for reinforcement learning to improve over time, both for individuals and at the group level [58].

The performance of reinforcement learning algorithms is enhanced when combined with machine learning algorithms that can extract and interpret multicomponent contextual information [59, 60]. With machine learning, reinforcement learning algorithms can “learn” the most effective action to produce a desired outcome based on the combination of a range of complex contextual factors [58], such as individual preferences, sleep-wake patterns over time, working schedules, and fatigue levels. Before the development of advanced AI methods, reinforcement learning algorithms were limited to simpler associations between only a few contextual variables [59]. The ability for reinforcement learning to interpret a wider range of contextual variables and provide progressively more personalized advice [58, 59, 61] is particularly relevant for people working nonstandard hours, whose routines vary greatly with shifting schedules.

Natural language processing and LLMs also present an exciting opportunity for a JITAI tailored to individuals working nonstandard hours. Natural language processing allows computers to interpret and respond to freely written and spoken human text and speech [62]. This facilitates the ability for digital assistants, also known as chatbots or conversational agents, to interact with humans in a more natural way [62]. Until recently, natural language processing assistants have also required their responses to be preprogrammed. However, LLMs, such as ChatGPT and Google Gemini, have significantly advanced the potential of natural language processing [60]. LLMs can generate “intelligent” responses to human text and speech based on vast datasets and do not require human programming [63]. This type of delivery method for a digital health behavior change intervention allows users to access personalized support at any time of day by “reaching out” or “chatting” with the assistant. LLM-based digital assistants could extend this further by providing greater personalization, improved efficacy, and true 24/7 access, making them well suited for a JITAI for individuals working nonstandard hours.

**Figure 1:**
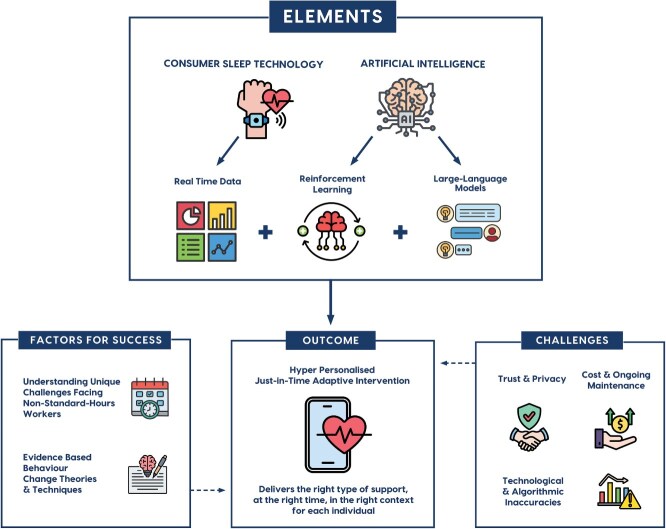
Elements, necessary factors for success, challenges, and outcomes of a JITAI combining AI and consumer sleep technology.

By combining reinforcement learning’s ability to optimize interventions over time with LLMs’ conversational interface, a JITAI could deliver the right sleep advice at the right time and context for nonstandard-hour workers. This approach has already shown promise in physical activity [64]. For example, the MoveMentor digital assistant aims to improve physical activity levels in office workers by delivering personalized nudges based on activity patterns and preferences, supported by an LLM-driven conversational interface through which users can ask questions. To deliver tailored physical activity advice, MoveMentor integrates data from wearable consumer activity trackers, reinforcement learning, machine learning, natural language processing, and LLMs [65]. User focus groups embraced the concept, but emphasized the need for customization in areas such as frequency of nudges and data sharing [65]. These findings highlight the potential for a hyper-personalized JITAI that integrates sleep tracking, reinforcement learning, and LLMs to provide real-time, tailored support (see Figure 1 for visual concept).

A JITAI that combines wearable consumer sleep tracking devices with reinforcement learning and LLMs could overcome limitations of one-size-fits-all solutions and set a new standard for health and safety interventions in variable work environments. Wearable consumer sleep trackers allow access to real-time contextual data [45], while reinforcement learning algorithms enhance the type and timing of sleep advice based on associations across a range of complex contextual variables [59]. LLMs allow for the delivery of sleep advice via a conversational interface where users can “reach out” and receive personalized advice at any time [63]. Together, these elements could provide a highly personalized JITAI capable of adapting to each individual nonstandard-hour worker’s unique sleep/wake time, working schedule, challenges, and individual preferences.

### Necessary factors for a successful JITAI underpinned by machine learning and consumer sleep technology

#### Applying behavior change theories and techniques with proven efficacy

There is a significant gap between the technological capabilities of JITAIs and evidence regarding the types of behavior change theories and techniques that are most effective when implemented within these systems [33]. While JITAIs can deliver highly personalized, real-time interventions, limited evidence exists to guide the optimal theoretical and behavioral foundations underpinning these features. Addressing this gap is essential for updated JITAI models to ensure they are not only technically advanced but also grounded in behavior change approaches with demonstrated effectiveness. Among the limited number of evidence-based JITAIs that use wearable consumer devices and machine learning to inform behavior change [64–66], developers have drawn on theories such as Self-Determination Theory [67], Social Cognitive Theory [68], the COM-B Model [69], and Nudge Theory [70]. Behavior change techniques such as self-monitoring, gamification, goal setting, personalized feedback [71], and use of online social networks [72] are effective in mobile health behavior change interventions and well suited to a JITAI using machine learning and LLMs. Training a JITAI with reinforcement learning and LLMs, underpinned by evidence-based theories and techniques, provides a strong foundation for delivering tailored behavior support to specific demographics before becoming further personalized.

#### Understanding unique contexts and challenges facing nonstandard-hour workers

For a machine learning–based JITAI to improve sleep in nonstandard-hour workers, it is essential that its algorithms take the unique challenges and contexts that these workers face into account. Interventions should use guidelines specific to nonstandard-hour workers rather than standard sleep hygiene recommendations [28]. A machine learning–based JITAI for this group is a novel concept, and understanding factors such as user interface, types and timing of nudges, and individual preferences is also important. One way to achieve this is through cocreation with nonstandard-hour workers themselves, alongside relevant experts and academics. In the context of public health, cocreation is defined as “collaborative public health intervention development by academics working alongside other stakeholders” [73]. Applied here, cocreation could help to identify barriers and facilitators relating to their irregular schedules and guide developers on the most appropriate design features for such an intervention.

### Challenges

#### Trust

Public hesitancy and distrust toward machine learning technology [74] present a challenge, as the success of AI-driven health behavior change interventions depends on people’s willingness to trust and engage with them [75]. In addition to distrust in machine learning, a key challenge for hyper-personalized interventions is the privacy–personalization paradox [76]. The privacy–personalization paradox describes the notion that the more personalized a digital intervention becomes, the more data participants must share, which often raises privacy concerns [76]. Although the privacy–personalization paradox is recognized as a barrier, it can be mitigated by building trust, ensuring security, and demonstrating direct user benefit [74, 75, 77]. For example, in focus groups exploring a machine learning–based digital assistant for physical activity, participants raised privacy concerns but generally felt the personalization benefits outweighed the risks [65]. Overall, literature suggests that trust is essential to the success of a machine learning–based JITAI and that users are willing to share personal data when the benefits are clear and outweigh the risks [74, 75, 77]. Trust may also be strengthened through cocreation with end users.

#### Technological and algorithmic inaccuracies

Technological and algorithmic inaccuracies pose a further challenge to the success of an AI-based JITAI using wearable consumer sleep tracking devices to improve sleep of individuals working nonstandard hours. Wearable consumer sleep tracking devices commonly overestimate total sleep time [78], with many widely used devices misclassifying sleep onset latency (SOL) and wake after sleep onset (WASO) as sleep [79, 80]. When data received from wearable consumer sleep tracking devices are inaccurate, AI algorithms may provide sleep advice that is unhelpful. For example, if a wearable consumer sleep tracking device misclassifies significant portions of WASO or SOL as sleep, it may appear that an individual has slept efficiently for a sufficient duration. In this case, an AI-driven JITAI may avoid prompting the user to have a nap or wind down early, which is not conducive to improved sleep health. Providing sleep advice driven by inaccurate data also risks harm to users [81]. Studies have shown that participants given “sham” negative feedback regarding their previous night’s sleep via wearable sleep trackers displayed increased sleepiness and decreased alertness the following day [81]. Inaccuracies in data obtained through wearable consumer sleep tracking devices informing JITAIs must be considered to reduce the likelihood of unhelpful sleep advice being delivered to users. A potential mitigation strategy may be for developers to use data that are more accurate than measures of WASO or SOL to inform JITAIs, such as rolling averages of sleep duration and quality over multiple days, sleep opportunities, or shift schedule information. Importantly, reliability of wearable-derived metrics improves with additional monitoring days; establishing minimum monitoring windows is therefore a key design decision when using rolling averages to inform JITAI logic (e.g., minimum days required for reliable wearable outcomes [82]. Many wearable consumer sleep tracking devices are also approaching accuracies in sleep measurement comparable to clinical sleep technologies [38, 39]. Thus, the challenge of inaccuracies in data obtained through these devices may be less significant than suggested by some previous literature, and is likely to be mitigated as more wearable consumer sleep tracking devices approach clinical-grade accuracies in measuring sleep.

In addition to technological inaccuracies, AI algorithms are also prone to inaccuracies if not developed using high-quality, diverse datasets [83–85]. The ability for AI-driven JITAIs to provide highly tailored, context-dependent sleep advice to those working nonstandard hours relies heavily on their development with data that is representative of the heterogeneity in their working schedules [84]. Algorithms developed using small or biased datasets risk the potential for JITAIs to provide poorly timed sleep advice. For example, if a JITAI’s AI algorithms are developed using datasets that underrepresent night shift workers, it may suggest these workers avoid sleep during the day, which is necessary for these workers to achieve sufficient sleep duration [86]. Ensuring that AI algorithms used in JITAIs are developed using large, high-quality, diverse datasets is essential to success in delivering contextually appropriate sleep advice to all nonstandard-hour workers [84, 85].

#### User engagement and compliance

Achieving sufficient levels of user engagement and compliance is essential for a machine learning–based JITAI to be successful in changing behavior and may pose a challenge for developers. Digital behavioral interventions are often associated with high levels of attrition and declining participant engagement [87] and compliance over time [88]. For example, a study assessing user compliance with digital sleep diaries completed via a mobile app found high user compliance during the first 2 months of the study period, before significantly decreasing throughout the third month [88]. If user engagement and compliance with a machine learning-based JITAI are not sufficient to achieve behavior change, it may be no more effective than traditional sleep interventions. Despite the many examples of declining user engagement and compliance with digital behavioral interventions over time, literature has identified a number of factors shown to mitigate this challenge [33, 89]. Personalization [89], timely advice, and contextually appropriate advice have been recommended to increase engagement levels and reduce attrition rates in digital behavioral interventions [33]. Due to the contextually adaptive and highly personalized nature of machine learning–based JITAIs using wearable consumer sleep tracking devices, user engagement and compliance levels are likely to be sufficient. However, as with any untested intervention, improvements in user engagement and compliance rates will not be clear until additional research has been conducted.

#### Cost, accessibility, and feasibility

For a machine learning–based JITAI to be accessible to nonstandard-hour workers, a major consideration is the cost of a wearable sleep tracker accurate enough to inform prompts or nudges. Consumer devices priced around $100–$350 USD have been found to be comparable to research-grade actigraphy for measuring total sleep duration [39]. These devices would therefore be sufficient to inform nudges in behavioral sleep interventions. Although this remains a notable cost, it is far lower than individual counseling, the gold-standard intervention for hyper-personalized sleep advice. In addition to the cost of wearable consumer sleep tracking devices, the development and maintenance of a machine learning–based JITAI must also be considered. Training algorithms require large datasets over extended periods, creating a substantial upfront cost [56]. However, these costs are likely to be outweighed by the potential health and economic benefits of a scalable intervention. Insufficient sleep is estimated to cost $411 billion annually in the United States [91], $50 billion in the United Kingdom [91], $21.4 billion in Canada [90], and $26.2 billion in Australia, largely due to lost productivity [91]. Given that nonstandard-hour workers represent a large proportion of the workforce, improving their sleep through JITAIs could generate significant economic gains. Maintenance is another major expense, including software updates, troubleshooting, and technical support [92]. For example, the annual maintenance cost of an AI-based clinical health intervention has been estimated at around $20 000 USD per year [92]. This raises the question of who is responsible for ongoing costs, as following development and validation, they likely fall beyond the responsibility of researchers. Organizations employing nonstandard-hour workers may need to view this investment as part of supporting safety and productivity.

## Conclusion

The rapid advancement of consumer sleep tracking devices, now achieving accuracies once limited to clinical technologies, together with machine learning technologies such as reinforcement learning, LLMs, and natural language processing, present an exciting opportunity for JITAIs targeting health behaviors. This integration is particularly relevant for nonstandard-hour workers, who face irregular schedules and associated health and safety risks. A machine learning–driven JITAI informed by consumer-grade wearable sleep trackers and tailored to this group has the potential to overcome the limitations of current one-size-fits-all approaches and establish a new standard for personalized sleep, safety, and productivity interventions. For such interventions to be effective, however, they must also be grounded in appropriate behavior change theories, address the unique challenges of nonstandard-hour work, and carefully consider issues of trust, cost, and accessibility.

## Data Availability

N/A
